# The relationship between mind wandering and reading comprehension: A meta-analysis

**DOI:** 10.3758/s13423-022-02141-w

**Published:** 2022-07-15

**Authors:** Paola Bonifacci, Cinzia Viroli, Chiara Vassura, Elisa Colombini, Lorenzo Desideri

**Affiliations:** 1 Department of Psychology, University of Bologna, Viale Berti Picaht 5, Bologna, Italy; 2 Department of Statistical Sciences, University of Bologna, Via Belle Arti 41, Bologna, Italy

**Keywords:** Mind wandering, Reading comprehension, Meta-analysis, Text type

## Abstract

Mind wandering (MW), a shift of attention away from external tasks toward internally generated thoughts, has been frequently associated with costs in reading comprehension (RC), although with some contrasting results and many reported potential intervening factors. The aim of the meta-analysis was to evaluate the relationship between MW and RC, considering the role of participants’ and text’s characteristics, as well as methodological issues in the measurement of the two constructs. From a set of 25 selected full texts (73 correlation coefficients), pooled correlation (*r* = −0.21) revealed a negative significant relationship. Using trait-based questionnaires to assess MW compared with online probes resulted in an average significant change of 0.30 in the correlation between MW and RC, leading to a null correlation. A significant effect of age was also found, with more negative correlations with increasing age. None of the other moderating variables considered (i.e., language, text type, text length, RC assessment, text difficulty, text interest, and working memory) resulted in a significant effect. From the present meta-analysis, we might suggest that MW and RC are partially overlapping and vary, within a *swing effect*, in relation to a set of shared factors, such as working memory, interest, and text length. There might also be side-specific factors that drive the movement of primarily one side of the swing, and future research should further consider the role of individual differences in RC. Implications for research and educational settings are discussed.

## Introduction

Mind wandering (MW) can be broadly defined as a shift in the contents of thought away from an ongoing task and/or from events in the external environment to internally self-generated thoughts and feelings (Smallwood & Schooler, 2015). Estimates suggest that the tendency for the mind to stray from the “here and now” in favor of task-unrelated thoughts constitutes as much as 50% of our waking hours in healthy adults (Killingsworth & Gilbert, 2010). In this view, it can be argued that MW per se is not a dysfunctional process but rather an essential phenomenon for human experience.

Over the past decade, an increasing number of theories have tried to capture the unique characteristics of MW in an attempt to define it. Actually, MW refers to a wide range of experiences that vary in content, intentionality, and relationship between activities and external stimuli (Seli et al., 2018). Four theories, in particular, have been put forward (Irving et al., 2020). The standard approaches to MW define this phenomenon as either *task-unrelated thought* (thought disengaged from one’s primary task) and/or *stimulus-independent thought* (i.e., thought decoupled from perception; Smallwood & Schooler, 2006; Smallwood & Schooler, 2015). An alternative approach, in contrast, refers to MW as *unintentional thoughts* that arise independently of conscious intentions (McVay & Kane, 2010; Watzl, 2017). Finally, a fourth approach further conceives MW as a *dynamic and unguided thought* that floats from topic to topic over time (Christoff et al., 2016; Irving, 2016; Irving & Thompson, 2018; Mills et al., 2018). Instead, Irving et al. (2020) emphasize that the dynamic through which thoughts unfold over time is the core feature of MW; but Seli et al. (2018), suggest replacing the idea of “core features” of MW by a family-resemblances approach that might better account for the heterogeneity and complexity of the phenomenon.

Despite the differences in the views and definitions of MW, both positive and negative influences of MW on cognitive performance have been reported in the literature (for a review, see Mooneyham & Schooler, 2013). Some authors, for example, suggested that MW might support adaptive functions such as planning, creative thinking, problem-solving, creative incubation, allowing dishabituation, and relieving tedium (Baird et al., 2012; Baird et al., 2011; Mooneyham & Schooler, 2013; Ruby et al., 2013; Stawarczyk et al., 2011). However, several studies failed to replicate a relation between problem-solving/creativity and mind wandering (Smeekens & Kane, 2016; Steindorf et al., 2021), particularly when considering MW contents and using probes instead of retrospective reports (Murray et al., 2021). Further, eventual benefits brought about by MW seem to be counterbalanced by evident costs for cognitive performance (Mooneyham & Schooler, 2013). For instance, current evidence suggests that the tendency to engage in MW might have detrimental effects on performance when it occurs during demanding cognitive tasks tapping working memory, general intelligence, and sustained attention (Cheyne et al., 2009; Franklin et al., 2011; McVay & Kane 2012; Mrazek et al., 2012; Reichle et al. 2010; Schooler & Schreiber, 2004; Smallwood, 2011; Smallwood et al., 2008; Smilek et al., 2010).

Of particular relevance for the scope of the present paper is the evidence suggesting that MW might negatively impact academic achievement by reducing students’ comprehension of written texts (Smallwood, 2011). Furthermore, given the importance of text comprehension as a fundamental prerequisite to cope with demands of daily life and achieve important educational and personal life goals (Meneghetti et al., 2006), it can be argued that a deeper understanding of the mechanisms associated with proficient or poor comprehension skills in the general population is central for research and educational settings. Accordingly, the present paper aims to provide a comprehensive review and meta-analysis of the studies reporting relationships between MW rates and reading comprehension (RC) performance in adolescents and young adults.

RC is a multifactorial process, and many theoretical models have been developed to explain the cognitive processes involved. As suggested by the Simple View of Reading (Hoover & Gough, 1990), RC can be seen as the product of decoding skills and listening comprehension skills. This model has guided research in opaque (Kendeou et al., 2009) and transparent orthographies (Bonifacci & Tobia, 2017; Tobia & Bonifacci, 2015). However, from a broader perspective, many different subcomponents interact to allow the reader to reach a deep understanding of the text. According to the construction-integration model proposed by Kintsch and Rawson (2005), text comprehension involves three levels of representation hierarchically ordered: the lexical level, the propositional level, and the situational level. The lexical level requires extracting the perceptual information from the page and finding the lexical meaning of letters and words in the working memory. The propositional level requires organizing words into propositions (e.g., understanding the meaning of sentences or paragraphs in the text). Finally, the situational level, which is the highest and more complex level, requires going beyond the explicit content of the text and integrating it into a global context through access to readers’ previous knowledge and inferential processes. Such an articulated and multi-component process requires a high attention rate to have a constant connection between bottom-up representations deriving from the text, and top-down representations, deriving from the reader (Kintsch, 2005).

According to Smallwood (2011), when we focus on the information coming from our perceptual systems, our attention is coupled to the continuous flow of sensory information. In contrast, when we think about our internally generated thoughts and feelings, our attention is *decoupled* from the external world. In the latter case, the attention to our internal thoughts and feelings implies insensitivity to external perceptual inputs. Such decoupling of attention between internally generated thoughts not related to the task at hand (i.e., MW) and the attention towards external information may explain the negative influence of MW for comprehension performance during reading, for which the occurrence of MW episodes while reading is associated with deficits in representation at the lexical, propositional, and situational levels of the text (Smallwood, 2011).

Specifically, the “cascade model of inattention” (Smallwood, 2011) proposes that the decoupling of attention causes a reduction in perceptual information processing—both auditory and visual information. In turn, this reduction leads to an incomplete execution of processes relevant to stimulus comprehension, and at the same time, it compromises the performance of tasks such as perceptual identification, target identification, and encoding (Smallwood, 2011; Smallwood et al., 2007). Encoding errors prevent the opportunity for rich episodic encoding from happening, leading to impaired performance. According to this model, engaging in MW during reading causes a cascade effect on performance. In particular, since RC requires the information to be processed in an orderly fashion (Kintsch & Rawson, 2005), states of decoupled attention (i.e., MW) may lead to degraded perceptual representations. This condition will prevent detailed lexical processing, which in turn will damage the creation of propositions. Furthermore, the absence of bottom-up information processing hinders the reader’s ability to create a complex propositional and situational model of the text.

Stemming from the seminal work of Smallwood et al. (2007; Smallwood, 2011), research aimed at investigating whether MW is associated with poor RC performance during reading has produced mixed results. While several studies report a negative impact of MW on RC (e.g., Feng, et al., 2013; Reichle et al., 2010; Smallwood et al., 2008; Smallwood et al., 2008; Smilek et al., 2010; Unsworth & McMillan, 2013), others have failed to observe such negative association (e.g., Broadway et al., 2015; Desideri et al., 2019; Varao-Sousa et al., 2013; Zhang et al., 2019).

Further, contradicting results were found within studies that assessed the relationship between MW and RC under different conditions and/or through different sampling measures of MW. For example, Varao-Sousa et al. (2013) reported significant negative correlations between MW and RC in the reading silently but not in the reading aloud condition. Other text characteristics that might impact MW are text type and text length (Feng et al., 2013; Forrin et al., 2019). Regarding text type, McVay and Kane (2012) found a negative relationship between MW measures and both types of narrative and expository text comprehension; however, less evidence has been collected on a direct comparison between different types of texts and heterogenous results within each category have been reported. As far as text length is concerned, evidence suggests that longer texts might generate higher rates of MW (Forrin et al., 2021; Forrin et al., 2018, 2019). These authors suggest that individuals may tend to disengage their attention from passages with long text sections because they appear to be more demanding than passages with shorter sections. Unsworth and McMillan (2013) found that both reader’s interest in the text’s content being read and motivation are important determinants of MW while reading. Specifically, participants who indicated that they were not interested in the topic of the text also reported more MW than individuals who were interested in the topic. Furthermore, individuals who stated they were more motivated to read the text and perform well reported less MW than individuals who indicated that they were not motivated.

To complicate the matter further, factors that are thought to influence MW during reading have also been found to affect RC performance. For instance, previous research analyzed comprehension differences between narrative versus informative texts, which demand different cognitive skills (Eason, et al., 2012) and strategies for answering (Tobia & Bonifacci, 2020), and it has been suggested that narrative texts might be easier to comprehend than expository texts (Best et al., 2008; Yildirim et al., 2010). In addition, reading assessment might also indirectly impact RC, as it was found that is decoding, not oral comprehension, that accounts for most of the variance in tests that used cloze tasks to assess RC, whereas the reverse holds for tasks with open questions (Francis, et al., 2005; Keenan et al., 2008; Tobia & Bonifacci, 2015). Reading processes are also related to orthographic transparency (Seymour et al., 2003), and reading models developed and tested on a single language could be misleading (Share, 2008). When the reading process is more challenging, as for students with dyslexia, text-to-speech reading application might reduce MW compared with the self-paced reading condition (Bonifacci et al., 2022). As for MW, also for RC, significant effects of reading interest (Babbitt Bray & Barron, 2004) and motivation (Wigfield et al., 2016) have been found. A greater interest may lead to placing more attentional resources on the text (Hidi, 2001), which in turn may improve the reader’s retention of the contents of the text and allow a deeper elaboration of it, leading the reader to have a better comprehension and a richer mental model of the text (Kintsch, 1988).

At a cognitive level, working memory skills are thought to play a significant role in both RC (De Beni, et al., 1998; Follmer, 2018; Palladino et al., 2001) and MW. Working memory serves as a buffer for integrating and establishing the coherence of different text parts, allowing to keep relevant information and discard irrelevant information from the buffer. This process needs to be repeated during reading to construct text meaning and coherence. Working memory has been found to be negatively associated with MW (McVay & Kane 2009; Randall et al., 2014; Unsworth and McMillan; 2013) since individual differences in working memory capacities appear to stem in part from momentary failures of conscious thought control. Interestingly, the study by McVay and Kane (2012) showed that the association between working memory and RC is partially mediated by MW rate, suggesting that control over thought content is one of the pathways through which variation in working memory capacity influences reading ability. In addition, Unsworth and McMillan (2013) evidenced that, together with working memory, interest and motivation influence the likelihood of MW which, in turn, mediate RC skills.

Finally, the methodological approach used to measure rates of MW and the associated experimental paradigms varied considerably between studies. Typically, to examine the effect of MW on reading, a self-paced paradigm is used where participants are required to read a word, sentence, or paragraph of text at a time and are randomly probed with questions inquiring if they were on task or off task (Mooneyham & Schooler, 2013). Some studies have used online self-report of mindless reading (e.g., Kopp et al., 2015); that is, as soon as the participant realizes that his or her mind is wandering, he or she presses a button to signal that he or she had been engaged in mindless reading (Nguyen, et al., 2014). In other cases, posttask self-reports about the extent of MW experiences during the prior task were used (e.g., Sanders et al., 2017; Soemer et al., 2019). Overall, these studies show that MW during reading leads to slower reading times, longer average fixation duration, and the absence of the word frequency effect on gaze duration (Foulsham et al., 2013), with a negative influence on the comprehension of difficult texts (Feng, et al., 2013). Although real-time assessment is considered the most reliable procedure to understand the phenomenon of MW, studies have shown that reports of MW assessed via thought probes during a task are consistently and significantly correlated with posttask self-reports (e.g., Zhang et al., 2019) and dispositional (i.e., trait-based) measures of MW obtained by questionnaires (e.g., Smallwood et al., 2003; Smallwood et al., 2006), even in young adults and adolescents (Stawarczyk et al., 2014; Varao-Sousa & Kingstone, 2019). Researchers have also developed methodologies for measuring MW through the detection of eye movements (e.g., Mills et al., 2020).

In sum, results from multiple studies over the last decade generally support the idea that MW may negatively impact RC, as zoning out while reading is supposed to progressively degrade the information needed to build an efficient situational representation of the text (e.g., Smallwood, 2011). It remains an open question, however, (a) the magnitude of the association between MW and RC and (b) whether the mixed results available on the relationship between MW and RC may be associated with the influence of potential intervening factors, such as MW assessment procedures (probes, online self-report, posttest self-report, trait measures, eye-gaze measures), RC measures (multiple-choice, open-ended, true–false questions), text type (expository vs. narrative), text length, and text language (e.g., transparent or opaque languages). Further, participants’ characteristics, such as age (Mrazek et al., 2013), and dispositional traits, such as topic interest or perceived difficulty of the text, might play a role in the relationship between MW and RC. Finally, cognitive traits such as working memory capacity have been accounted to play a role in both MW and RC (Unsworth, & McMillan, 2013). More generally, the role of individual differences needs to be adequately tested both in MW (Robison et al., 2020) and in RC (Schindler, & Richter, 2018).

To date, there are no meta-analyses that systematically investigated the strength of the association between MW and RC as well as explored the influence of relevant intervening factors on such a relationship. Recently, D’Mello and Mills (2021) reported on a mini-meta-analysis combing results from the reading results in Randall et al.’s (2014) study and 25 effects from studies conducted in their lab. They found a weighted mean correlation between MW and RC of −0.31. However, as underlined by the same authors, this revision did not include a systematic review of the literature, and therefore, in the present study, we aim to address this issue. Our first aim was to identify studies investigating the effects of MW on RC in adolescents and adults to estimate the magnitude (and direction) of the relationship between MW and RC. Our secondary aim was to identify potential moderators of the association between MW and RC, including assessment procedures of MW and RC, participant’s age, text language (transparent vs. opaque), text length and difficulty, interest with the topic, and working memory.

## Method

**Fig. 1:**
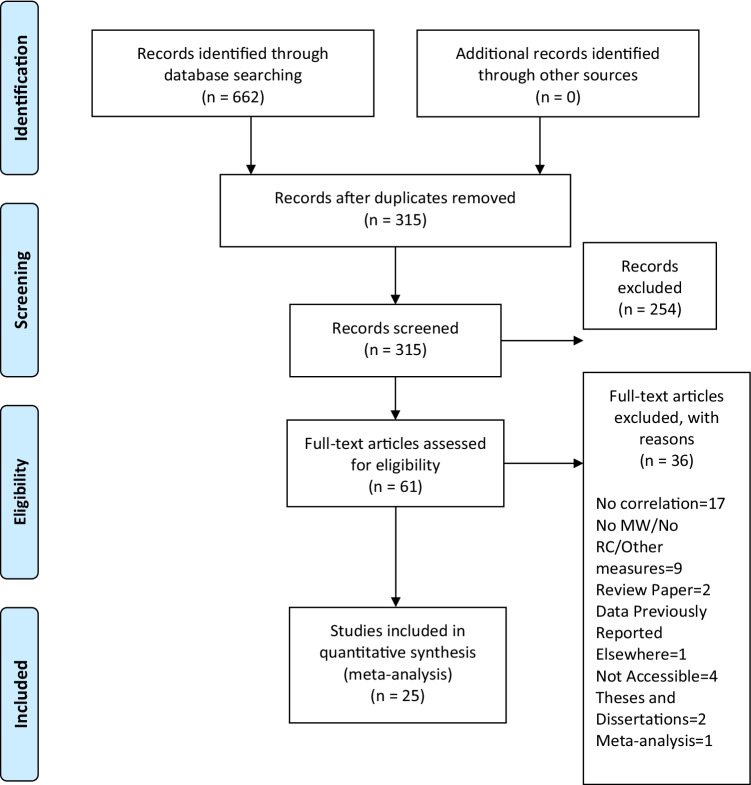
Overview of the literature search process

A systematic search was conducted following the Preferred Reporting Items for Systematic Reviews and Meta-Analyses (PRISMA) reporting guideline recommendations (Moher et al., 2010) to identify studies reporting on the influence of MW on RC in healthy adolescents or adults. The search was performed using the following academic databases: Web of Science, Scopus, PubMed, EBSCO (i.e., PsychInfo, PsychArticles, and ERIC). The database search was conducted between January 2020 and March 2020 and was restricted to English-language, peer-reviewed journals. Search terms related to MW (“mind-wandering,” “daydreaming,” “mindless,” “mind pops,” “stimulus-independent thoughts,” “task unrelated thoughts,” “self-generated thoughts,” “zoning out”) were combined with search terms related to RC (“reading comprehension,” “reading,” “comprehension”). Figure 1 illustrates the search process and outcome. Grey literature was not considered in the present meta-analysis. As suggested by Schmucker et al. (2017), although studies excluding grey literature might be likely to overestimate the treatment effects, current empirical research shows that this is the case only in a minority of reviews; further, publication bias might particularly affect specific research fields where there is need of publishing positive results more rapidly. In addition, grey literature is generally not peer reviewed, and the internal validity of unpublished data may be difficult to assess due to poor reporting of the trials, thus possibly increasing the risk of bias. Finally, although grey literature is an important resource for meta-analyses, there is little specific guidance and no accepted gold standard method for conducting rigorous gray literature searches (Paez, 2017).

Of the 314 article titles identified, through the evaluation of two independent coders (third and fourth authors), 61 were considered eligible for further analysis and fully read. Interrater agreement on abstract selection was 93.02%; in case consensus was not achieved, the first and last authors jointly reached a final evaluation. Full texts were included in the meta-analysis when studies (a) reported measures of MW and RC (e.g., studies with recall tasks were excluded), (b) involved school-aged participants (>10 years old) and/or adult (ages 18–40) skilled readers without any reported clinical condition, (c) reported correlation indexes (Pearson’s *r*) between MW and RC. For full-text selection, two independent coders (third and fourth authors) completed the first evaluation with an interrater agreement of 91.7%; if consensus was not achieved, the first and last authors jointly reached a final evaluation. The third and fourth authors firstly coded data reported in Table 1, then the first author conducted an independent coding of data, and the last author further checked all discrepancies with the original data set.

**Table 1:** Studies included in the meta-analysis

Study	Language	*N*	Age	Gender	Type of text	Text length (words)	RC Task	MW Assessment	Topic interest	Text difficulty	Working memory capacity
Al-Balushi & Al-Harty, 2015	Arabic	65	9th Graders	F(65)	Informative	1,635*	Multiple-choice	Probes			
Bixler & D’Mello, 2016 [a]	Mixed	178	20 (3.6)	F(111); M(67)	Informative	1,500	Multiple-choice	Probes			
Bixler & D’Mello, 2016 [b]	Mixed	178	20 (3.6)	F(111); M(67)	Informative	1,500	Multiple-choice	Probes			
Broadway et al., 2015	English	47	18.56 (0.14)	F(33); M(13)	Informative		Multiple-choice	Probes			
D’Mello et al., 2017 [a]	English	104	Under. Stud.		Informative	6,500	Multiple-choice	Eye gaze			
D’Mello et al., 2017 [b]	English	104	Under. Stud.		Informative	6,500	Multiple-choice	Eye gaze			
Desideri et al. 2019	Italian	272	17.23 (1.1)	F(133); M(139)	Narrative		Multiple-choice	Trait			
Franklin et al., 2011	English	49	19.2	F(23); M(26)	Narrative	5,000	Multiple-choice	Probes			
Franklin et al., 2014	English	74	20.2	F(48); M(26)	Narrative	5,000	Multiple-choice	Probes			
Kopp & D’Mello, 2016 [a]	English	51	28 (19-49)		Narrative	3,658	Multiple-choice	Probes			
Kopp & D’Mello, 2016 [b]	English	49	32 (18-54)		Narrative	3,658	Multiple-choice	Probes			
Kopp et al., 2015	English	140	20.2	F(88); (M(53)	Informative	5,700	Multiple-choice	Self-report online			
McVay & Kane, 2012 [a]	English	248	18-35		Narrative	8,000	True-false	Probes			−0.11
McVay & Kane, 2012 [b]	English	248	18-35		Narrative	8,000	True-false	Probes			−0.13
McVay & Kane, 2012 [c]	English	242	18-35		Informative	2,250	True-false & Multiple-choice	Probes			−0.02
McVay & Kane, 2012 [d]	English	242	18-35		Informative	2,250	True-false & Multiple-choice	Probes			−0.08
McVay & Kane, 2012 [e]	English	248	18-35		Informative	2,250	True-false & Multiple-choice	Probes			−0.07
McVay & Kane, 2012 [f]	English	242	18-35		Informative	2,250	True-false & Multiple-choice	Probes			−0.06
Mills & D’Mello, 2015 [a]	English	318	27		Informative	1,500	Multiple-choice	Probes			
Mills & D’Mello, 2015 [b]	English	318	27		Informative	1,500	Multiple-choice	Probes			
Mills et al., 2020 [a]	English	70	21.09 (2.9)	F(41); M(29)	Informative	6,500	Multiple-choice	Eye gaze			
Mills et al., 2020 [b]	English	70	21.09 (2.9)	F(41); M(29)	Informative	6,500	Multiple-choice	Eye gaze			
Mrazek et al., 2013 [a]	English	106	High Sch.	F(106)			Standard test	Probes			
Mrazek et al., 2013 [b]	English	106	High Sch.	F(106)			Standard test	Trait			
Mrazek et al., 2013 [c]	English	78	Middle Sch.	F(43); M(35)			Standard test	Probes			
Mrazek et al., 2013 [d]	English	78	Middle Sch.	F(43); M(35)			Standard test	Trait			
Noah et al., 2018 [a]	English	114	Univ. Stud.		Informative	515	Multiple-choice	Probes	−0.14	0.21	
Noah et al., 2018 [b]	English	114	Univ. Stud.		Informative	515	Multiple-choice	Probes	−0.35	0.26	
Noah et al., 2018 [c]	English	278	Univ. Stud.		Informative	515	Multiple-choice	Probes	−0.28	0.09	
Noah et al., 2018 [d]	English	278	Univ. Stud.		Informative	515	Multiple-choice	Probes	−0.04	0.03	
Phillips et al., 2016 [a]	English	96	AMT^1^		Informative	1,490	Multiple-choice	Probes	0.00		
Phillips et al., 2016 [b]	English	87	AMT^1^		Informative	1,491	Multiple-choice	Probes	−0.41		
Robison & Unsworth, 2015 [a]	English	121	19.9 (2.89)	F(149); M(92)	Informative		Multiple-choice	Probes	0.28	−0.17	−0.25
Robison & Unsworth, 2015 [b]	English	121	19.9 (2.89)	F(149); M(92)	Informative		Multiple-choice	Probes	0.36	−0.18	0.06
Robison & Unsworth, 2015 [c]	English	121	19.9 (2.89)	F(149); M(92)	Informative		Multiple-choice	Probes	0.44	−0.22	
Robison & Unsworth, 2015 [d]	English	121	19.9 (2.89)	F(149); M(92)	Informative		Multiple-choice	Probes	0.21	−0.1	
Robison & Unsworth, 2015 [e]	English	121	19.9 (2.89)	F(149); M(92)	Informative		Multiple-choice	Probes	0.34		
Robison & Unsworth, 2015 [f]	English	121	19.9 (2.89)	F(149); M(92)	Informative		Multiple-choice	Probes	0.46		
Robison & Unsworth, 2015 [g]	English	120	19.9 (2.89)	F(149); M(92)	Informative		Multiple-choice	Probes	0.11	0.05	
Robison & Unsworth, 2015 [h]	English	120	19.9 (2.89)	F(149); M(92)	Informative		Multiple-choice	Probes	0.27	0.04	
Robison & Unsworth, 2015 [i]	English	120	19.9 (2.89)	F(149); M(92)	Informative		Multiple-choice	Probes	0.33	0	
Robison & Unsworth, 2015 [j]	English	120	19.9 (2.89)	F(149); M(92)	Informative		Multiple-choice	Probes	0.13	0.01	
Robison & Unsworth, 2015 [k]	English	120	19.9 (2.89)	F(149); M(92)	Informative		Multiple-choice	Probes	0.28		
Robison & Unsworth, 2015 [l]	English	120	19.9 (2.89)	F(149); M(92)	Informative		Multiple-choice	Probes	0.32		
Sanders et al., 2017	English	93	20.1 (2.0)		Informative	1,039*	Open-ended	Self-report post			
Schurer et al. 2020	German	89	23.80 (0.365)	63% females	Informative	4,745*	Multiple-choice	Probes			
Smallwood et al., 2008	English	72	20	F(48); M(26)	Narrative	5,000	Multiple-choice	Probes			
Soemer & Schiefele 2019 [c]	German	214	24.23 (4.16)	F(176); M(40)	Informative	913–1,281	Multiple-choice	Probes	−0.30		
Soemer & Schiefele 2019 [a]	German	212	24.23 (4.16)	F(176); M(40)	Informative	913–1,281	Multiple-choice	Probes	−0.33		
Soemer & Schiefele 2019 [b]	German	213	24.23 (4.16)	F(176); M(40)	Informative	913–1,281	Multiple-choice	Probes	−0.30		
Soemer & Schiefele 2019 [d]	German	215	24.23 (4.16)	F(176); M(40)	Informative	913–1,281	Multiple-choice	Probes	−0.28		
Soemer & Schiefele 2019 [e]	German	216	24.23 (4.16)	F(176); M(40)	Informative	913–1,281	Multiple-choice	Probes	−0.22		−0.04
Soemer & Schiefele 2019 [f]	German	216	24.23 (4.16)	F(176); M(40)	Informative	913–1,281	Multiple-choice	Probes	−0.22		−0.15
Soemer & Schiefele 2020 [a]	German	179	23.35 (3.93)	F(155); M(25)	Informative	913–1,281	Multiple-choice	Probes	−0.30		−0.06
Soemer & Schiefele 2020 [b]	German	179	23.35 (3.93)	F(155); M(25)	Informative	913–1,281	Multiple-choice	Probes	−0.27		−0−13
Soemer & Schiefele 2020 [c]	German	179	23.35 (3.93)	F(155); M(25)	Informative	913–1,281	Multiple-choice	Probes	−0.26		−0.14
Soemer & Schiefele 2020 [d]	German	179	23.35 (3.93)	F(155); M(25)	Informative	913–1,281	Multiple-choice	Probes	−0.29		−0.08
Soemer & Schiefele 2020 [e]	German	179	23.35 (3.93)	F(155); M(25)	Informative	913–1,281	Multiple-choice	Probes	−0.22		
Soemer & Schiefele 2020 [f]	German	179	23.35 (3.93)	F(155); M(25)	Informative	913–1,281	Multiple-choice	Probes	−0.24		
Soemer et al. 2019 [a]	German	37	13.85 (0.65)	F(60); M(55)	Informative	426–438	Multiple-choice	Self-report post	0.22		
Soemer et al. 2019 [b]	German	37	13.85 (0.65)	F(60); M(55)	Informative	426–438	Multiple-choice	Trait			
Soemer et al. 2019 [c]	German	41	13.85 (0.65)	F(60); M(55)	Informative	426–438	Multiple-choice	Self-report post	0.26		
Soemer et al. 2019 [d]	German	41	13.85 (0.65)	F(60); M(55)	Informative	426–438	Multiple-choice	Trait			
Soemer et al. 2019 [e]	German	37	13.85 (0.65)	F(60); M(55)	Informative	426–438	Multiple-choice	Self-report post	0.19		
Soemer et al. 2019 [f]	German	37	13.85 (0.65)	F(60); M(55)	Informative	426–438	Multiple-choice	Trait			
Unsworth & McMillan, 2013 [a]	English	150	19.37 (1.49)	F(95); M(55)	Informative		Multiple-choice	Probes	−0.13		−0.14
Unsworth & McMillan, 2013 [b]	English	150	19.37 (1.49)	F(95); M(55)	Informative		Multiple-choice	Probes	−0.18		−0.13
Unsworth & McMillan, 2013 [c]	English	150	19.37 (1.49)	F(95); M(55)	Informative		Multiple-choice	Probes	−0.13		−0.17
Unsworth & McMillan, 2013 [d]	English	150	19.37 (1.49)	F(95); M(55)	Informative		Multiple-choice	Probes	−0.21		−0.21
Unsworth & McMillan, 2013 [e]	English	150	19.37 (1.49)	F(95); M(55)	Informative		Multiple-choice	Probes			−0.11
Unsworth & McMillan, 2013 [f]	English	150	19.37 (1.49)	F(95); M(55)	Informative		Multiple-choice	Probes			−0.22
Varao-Sousa et al., 2013 [b]	English	106	19.8 (2.15)		Informative	5,400	True–False	Probes	−0.20		
Varao-Sousa et al., 2013 [c]	English	106	19.8 (2.15)		Informative	5,401	True–False	Probes	−0.43		
Varao-Sousa et al., 2013 [e]	English	105	19.9 (1.63)		Informative	5,403	True–False	Probes	−0.36		
Varao-Sousa et al., 2013 [f]	English	105	19.9 (1.63)		Informative	5,404	True–False	Probes	−0.42		
Zhang et al. (2019) [a]	English	69	19.87 (2.33)	F(43); M(26)	Informative	1,050	Open-ended	Self-report online			
Zhang et al. (2019) [b]	English	69	19.87 (2.33)	F(43); M(26)	Informative	1,050	Open-ended	Self-report post			

In total, 25 articles were included in the meta-analysis (see Fig. 1), for a total of 73 correlation coefficients that emerged from multiple correlation indices reported in some studies.

### Data analysis

All correlation coefficients were entered independently in the case of papers with multiple studies or multiple correlations coefficients between MW and RC under different conditions. For studies reporting correlations involving latent factors (derived from confirmatory factor analysis), manifest-variable correlations were recovered from appendices included in the studies or by contacting the authors and asking for the original data sets (Soemer & Schiefele, 2019; Soemer et al., 2019). All cases of studies reporting latent factors were solved, and the final analysis included only manifest (Pearson’s *r*) correlation indices.

Each of the analyses was conducted in R (Version 4.1.1) using the *dmetar* package (Balduzzi et al. 2019; Schwarzer, 2007) with the Hunter–Schmidt method of pooling variance. Since studies vary with respect to several characteristics, including language, type of text, RC task, and MW assessment, some between-study heterogeneity can be expected, and it makes necessary to assume a random-effects pooling model. The between-study heterogeneity is measured with the Sidik–Jonkman estimator (Cuijpers, 2016). The adoption of random effect model was also needed to account for the methodological variability across studies (i.e., the within variability due to repeated participants designs; Hedges & Vevea, 1996). The random-effects model decomposes the variance with an additional component that captures extra-variability and calculates an adjusted random-effects weight for each study. The generic inverse-variance pooling method was also used to combine correlations from different studies into one pooled correlation estimate. When pooling correlations, we applied Fisher’s *z* transformation to obtain the weights for each study. Metaregression analyses were conducted to assess whether age, language type (i.e., transparent/opaque), text type (i.e., informative/narrative), text length (i.e., number of words), MW assessment (i.e., trait-based questionnaires, online probes, online self-report, posttest self-report, eye gaze), and RC procedures (i.e., open-ended, true–false, multiple-choice questions) could be considered intervening factors of the relationship between MW and RC performance. For identifiability reasons, in regression analysis with categorical predictors, such as some of our moderators, for each moderator (e.g., RC measures), a category (e.g., multiple-choice) was considered as reference, and its effect is incorporated in the model intercept. The other levels (e.g., open-ended and true–false) are measured in contrast with the reference one. In Table 2 the reference category is reported in footnotes.

**Table 2:** Test of moderators (significant effects in bold)

*Variable*	*Estimate*	*SE*	*z*	*p*	CI	
					Lower	Upper
Age	−0.016	0.005	−3.071	0.002	−0.027	−0.006
Text language	0.096	0.041	2.307	0.021	0.014	0.177
Text type	−0.072	0.063	−1.141	0.254	−0.195	0.051
Text length	0.000	0.000	0.885	0.376	−0.000	0.000
MW Eye-gaze*	0.022	0.080	0.278	0.781	−0.135	0.180
MW Self-report online*	−0.017	0.108	−0.162	0.871	−0.230	0.195
MW Self-report post*	0.013	0.083	0.168	0.867	−0.149	0.177
MW Trait*	0.305	0.069	4.370	0.000	0.168	0.4425
RC open-ended questions^	−0.059	0.100	−0.585	0.559	−0.255	0.138
RC True–false questions^	−0.021	0.072	−0.299	0.765	−0.162	0.119
Topic interest°	0.095	0.075	1.270	0.204	−0.052	0.242
Text difficulty°	0.226	0.218	1.036	0.300	−0.202	0.655
Working memory°	−0.241	0.398	−0.607	0.544	−1.020	0.538

*MW probes is the reference category

*^RC Multiple choice is the reference category*

*° In the metaregression correlation indexes between MW and these indexes were considered.*

Other potential mediators such as topic interest, text difficulty, and working memory capacity were reported in some studies but only as correlation values with MW (see Table 1). We included these indexes of correlation as candidate moderators of the relationship between MW and RC anyway. Eventually, publication bias was assessed through the Egger regression, the Begg test statistics and the Duval and Tweedie’s (2000) trim-and-fill procedure to check whether the pooled effect estimated in our meta-analysis could have been higher than the true effect size as we did not consider the missing studies with lower effects because they were never published (Rothstein et al., 2006). As suggested by Carter et al. (2019), no single meta-analytic method consistently outperformed all the others, and therefore reporting on a variety of methods is suggested as a valuable approach.

## Results

### Overview of included studies

Of the 25 papers identified, 15 papers (60%) included more than one study, for a total of 73 studies (i.e., correlation coefficients) eventually included in the meta-analysis. Thus, the 25 papers involved a total of 3,926 participants. Table 1 reports the details of the studies reported in each paper identified.

Studies were conducted mainly in English-speaking countries (*n* = 50; 68.5%). Other countries included Germany (*n* = 19; 26%), followed by Italy (*n* = 1; 1.4%) and Oman (*n* = 1; 1.4%). Two studies reported in one paper (Bixler & D’Mello, 2016) involved multiple languages (2.7%). When specific information on the language of testing was available, language type was coded as a transparent (Italian, German) or opaque language (English, Arabic), according to Seymour et al. (2003). Most of the studies (*n* = 62; 84.9%) employed informative written material, while the remaining studies either used narrative texts (*n* = 7; 9.6%) or did not explicitly report the type of text used (*n* = 4; 5.5%). The length of the texts used was reported in 38 (52%) of the 73 individual studies included in the analyses. On average, texts included *M* = 2,991.92 words (*SD* = 2,327.55 words). Across studies, RC was mostly assessed through multiple-choice questions (*n* = 61; 83.6%), while 4.1% included open-ended questions and 6.8% included true–false questions; others only reported the use of standardized tests without reporting the specific modality (5.5%). Online probes were most commonly used (*n* = 56; 76.7%) to assess MW, followed by 2.7% adopting online self-report, 6.9% posttest self-report, 8.2% trait measures, and 5.5% using eye-gaze measures. Associations between MW and topic interest, text difficulty, and working memory capacity were only reported in 41 (56%), four (5.47%), and 25 (32.5%) studies, respectively.

### Association between MW and RC

**Fig. 2:**
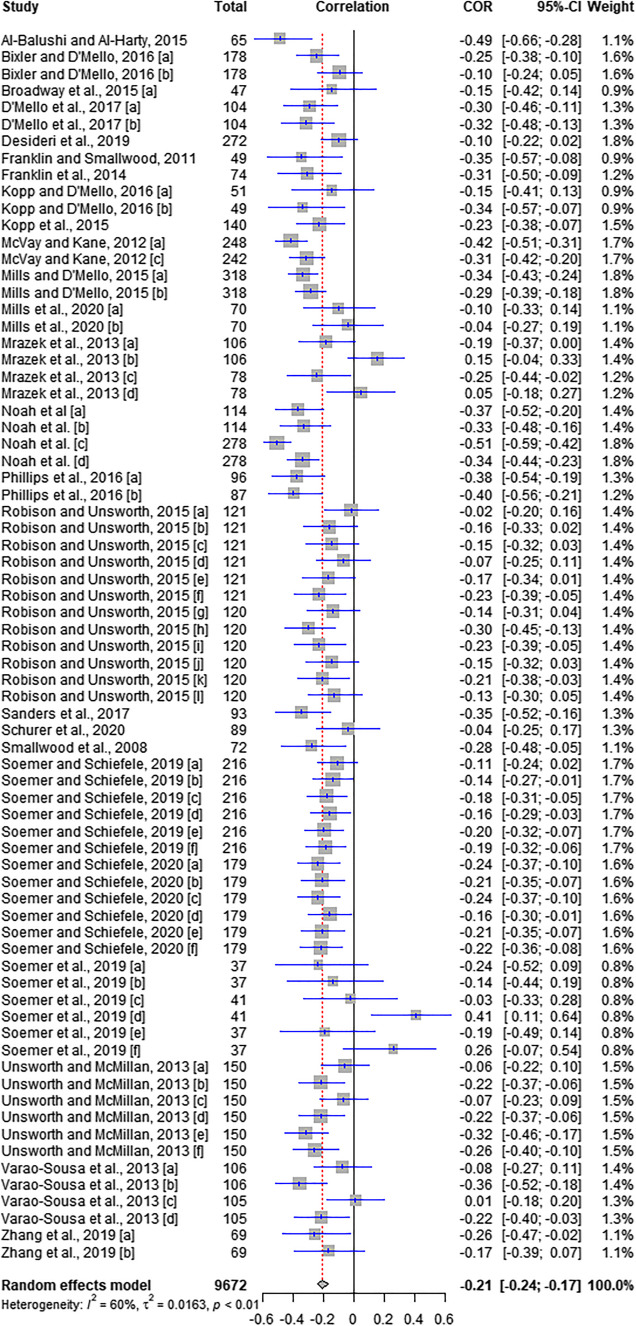
Effect of mind wandering on reading comprehension

In the first analysis, correlation indexes from 73 individual studies were considered (see Table 1). The *I*^2^ heterogeneity in this analysis is about 60%, supporting the use of the random effect model. As can be seen from the output (see Fig. 2), the pooled correlation in this data set is *r* = −.21 (*p* < .0001, 95% CI [−0.24, −0.1]), indicating a significant negative association between MW and RC—that is, people who tend to mind wander more often tend to exhibit lower reading comprehension. The same analysis on probed MW gives similar results, with a pooled correlation of *r* = −.23 (*p* < .0001, 95% CI [−0.26, −0.19]).

**Fig. 3:**
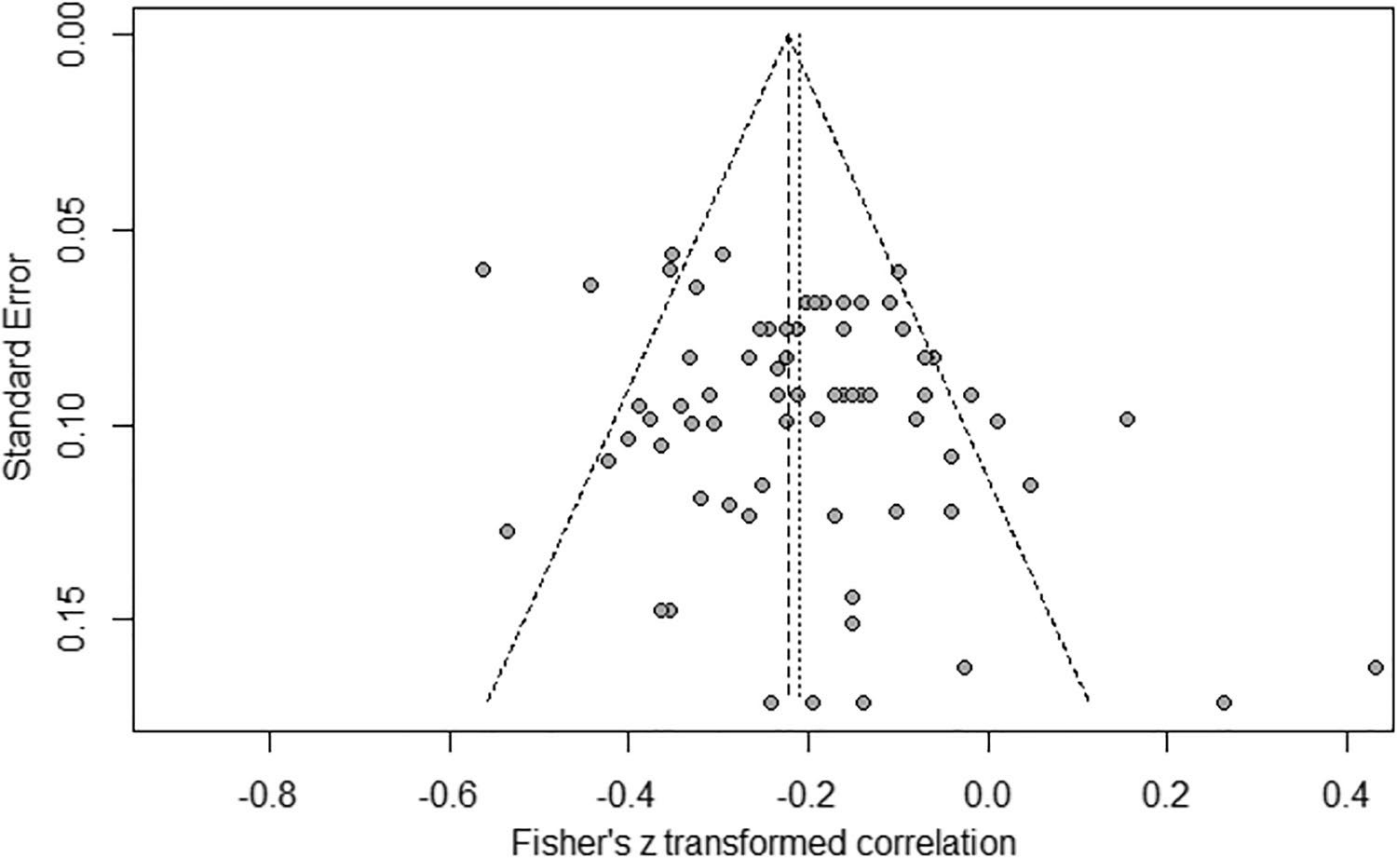
Trim-and-fill funnel plot for the data included in the meta-analysis

An additional sensitivity analysis was conducted to test the effect of studies with more than one experiment by merging them to their average correlation. This analysis yielded an overall correlation of −0.23 CI [−0.29, −0.18], which is consistent with the results of the complete data. Finally, to evaluate publication bias and confirm the robustness of these findings, we have applied the Egger’s regression, the Begg’s test statistics, and the trim-and-fill analysis. The funnel plot displayed in Fig. 3 shows an asymmetric pattern suggesting potential bias. The Egger’s regression has a *p* value at the limit of significance (*t* = 2.49, *df* = 71, *p* = .0152). Begg’s statistics test is not significant (*z* = 0.83, *p* = .4064), thus indicating the absence of bias. Finally, the trim and fill procedure added a total of 13 studies and produced a corrected correlation which is still significant (*r* = −.25, *p* < .0001, 95% CI [−0.29, −0.21]). To note, the outliers shown in Fig. 3 were balanced across the spectrum of possible *r* values (±1).

### Test of moderators

The effects of moderators considered that might affect the relationship between MW and RC are reported in Table 2. Specifically, we tested the effect of Age, Text Language type (Transparent vs. Opaque), Text type, Text length, MW assessment, Text difficulty, and RC assessment. Of these, age has a significant negative effect: when age increases by one year, the correlation decreases by −0.016 on average, thus bringing to a slightly higher negative correlation. Instead, the transparent language compared with the opaque one is associated with an increment of the correlation of an average of 0.096, which, however, was not fully significant (*p* = .021). Using trait-based questionnaires to assess MW with respect to online probes resulted in an average significant increase of 0.305 in the correlation between MW and RC, thus leading to an almost null correlation. None of the other moderating variables considered (i.e., text type, text length, RC assessment, text difficulty, text interest, and working memory) significantly affect the correlation.

## Discussion

The present study was aimed at conducting, for the first time with respect to previous literature, a comprehensive review and meta-analysis about the relationship between MW and RC and potential moderators.

First, we will discuss the strength of the relationships and the role of moderators. Then we will move to define a theoretical approach for interpreting the relationship and the related moderators considered in the light of previous literature and pointing out new perspectives and predictions.

From the meta-analysis on all selected studies, it emerged that the relationship is negative and significant (*r* = −.21), with a similar trend when considering only studies where MW was tested through on-tasks probes (*r* = −.23). According to widely used guidelines, the correlation can be generally considered in a low to moderate range (see, e.g., Cohen, 1992), as suggested by Delgado et al. (2018). As further suggested by Gignac and Szodorai (2016) for research addressing individual psychological differences, correlations ranging from .19 to .29 may be considered at the 50th percentile (“medium”). Furthermore, along with the magnitude of the effect, we argue that the meaning of this result should also be interpreted in light of the evidence that an effect size ranging from −0.21 to −0.32 is relevant in the RC field because it represents approximately two thirds of the yearly growth in RC during primary school (Luyten et al., 2017), and about one third of the effect of remedial reading interventions (Scammacca et al., 2015).

Notably, the strength of the association found in the present study mirrors that found in a previous meta-analysis (i.e., −0.24) conducted to assess the relationship between MW and adults’ performance in a wide set of cognitive tasks other than RC (e.g., interference control, sustained attention, visual search; Randall et al., 2014). On the counterpart, it was lower than what was found by D’Mello and Mills (2021), who merged results from their lab with those on reading reported in Randall et al.’s study that resulted in a correlation of *r* = −.31.

Overall, our results converge on previous findings that supported a negative relationship between MW and task performance in general (see, e.g., Randall et al., 2014) and RC in particular. Most notably, such relationship is relatively consistent across methodologies and potential moderators. In particular, a significant effect of age was found, with an increased negative relationship in adults, and an effect of MW assessment, with an almost null relationship between RC and MW when the latter is measured through trait-based measures instead of probes or post self-report. Finally, there was a marginal effect of language, but no effects of working memory, text difficulty, topic interest, and text type (narrative vs. informative) and text length were found. Considering age, the relationship between MW and RC became more negative with increasing age, with an estimated change of −0.016 per year. Of note, the studies included in our review involved both school-aged participants and/or adults; therefore, we did not consider the literature on aging, which usually reported that older adults tend to exhibit a lower rate of MW than younger adults (e.g., Krawietz et al., 2012). If MW tends to reduce over the years, it might be hypothesized that younger people tend to be more used to MW, whereas those who mind wander more frequently as adults might have more pronounced difficulties in inhibiting task-unrelated thoughts, leading to a stronger association between MW and RC.

Considering language, most studies were conducted on English texts and a minority on German, Arabic, and Italian, and language did not result in being a significant moderator. Finally, it has to be underlined that there were no studies where reading was required in a second language, and further research should address if the relationship between MW and RC changes in second language learners.

From a theoretical perspective, the significant correlation means that the two constructs are overlapping, at least in terms of covariance, since the relationship between MW and RC is relatively consistent and independent from a set of key moderators that previous literature highlighted as significant markers of either MW or RC. A set of shared factors that involve both text characteristics and individual differences in working memory might influence both the efficiency of RC and the occurrence of MW, but they seem not to affect the relationship *between* the two constructs; speculating that both MW and RC might modify their paths accordingly, at least to a certain degree, within an inverse relationship. Within this view, a unilateral causal model such as the cascade model of inattention (Smallwood, 2011) might not fully capture the nature of the relationship and shared underpinnings.

**Fig. 4:**
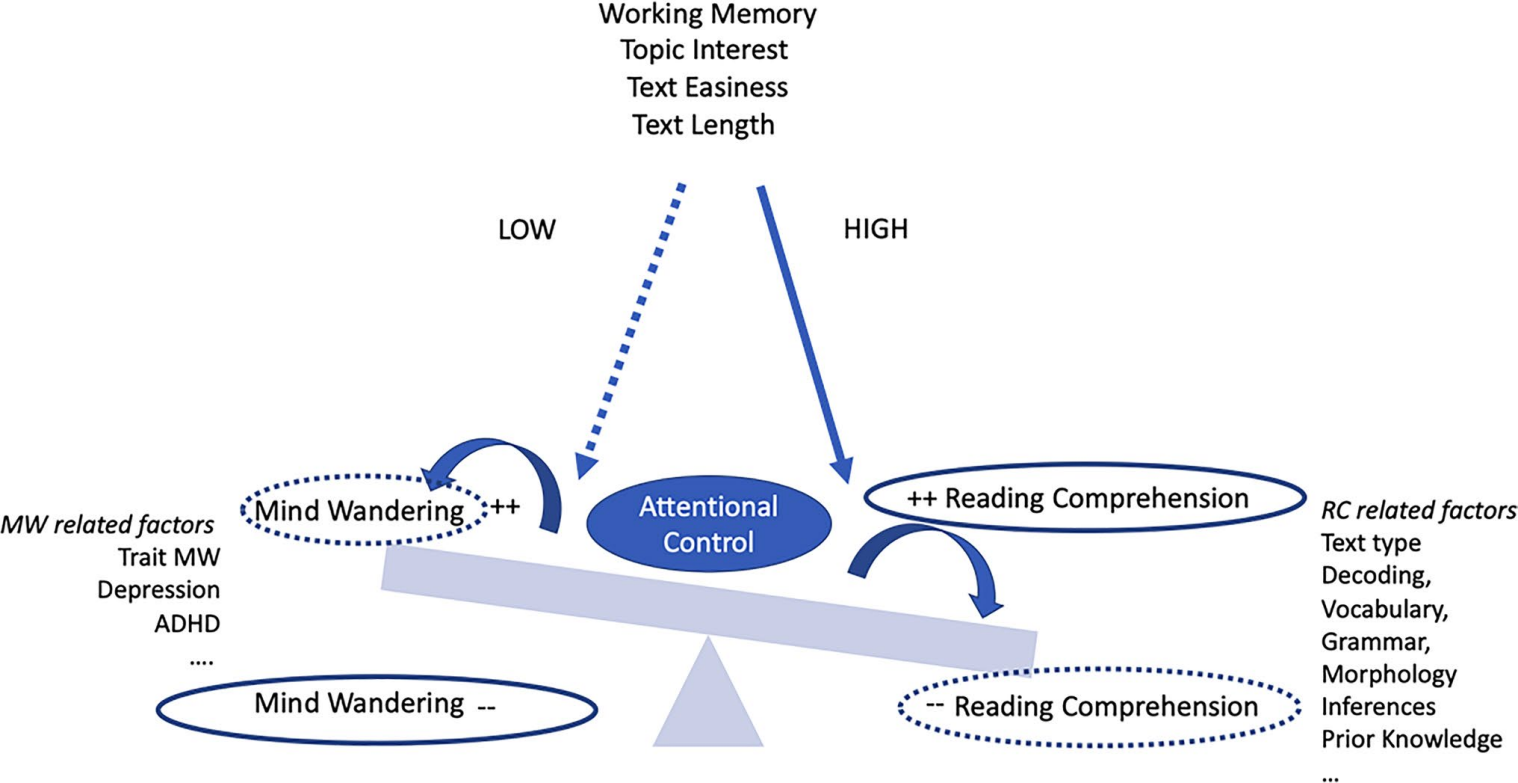
The image illustrates the shared and side-specific factors contributing to the relationship between mind wandering (MW) and reading comprehension (RC). The dashed line indicates that when the levels/performances in any of the shared factors such as working memory, topic interest, text easiness, or text length are low, higher levels (++) of MW should be observed together with lower RC performance (--). In contrast, the continuous line indicates that when the levels/performances in any of the aforementioned shared factors are high, lower levels (--) of MW should be observed along with higher RC performance (++). Other side-specific factors contributing to the relationship between MW and RC are listed at both sides of the swing and might indirectly impact on the counterpart. Individual differences in attentional control might further impact the extent to which the reader can adapt to the internal and external requests.

Therefore, we propose to interpret the relationship between RC and MW as an “up and down swing,” where when one dimension is up, the other goes down and vice versa, with the movement of the two sides of the swing (i.e., MW and RC) as determined by both shared and side-specific factors (see Fig. 4).

Based on the present meta-analysis, working memory, text difficulty, topic interest, and text length can be considered amongst the shared factors that equally impact MW and RC processes, possibly modulated by individual differences in attentional control that further impact the extent to which the reader can adapt to the internal and external requests. However, since MW and RC are two constructs that are not completely overlapping, we also recognize that there might be side-specific factors that drive the movement of primarily one side of the swing, such as mood for MW or decoding skills and vocabulary for RC. These side-specific factors, although exerting an influence mainly on one side of the swing (e.g., MW), might nevertheless have an indirect influence on the other side (e.g., RC).

Based on this theoretical approach, some further considerations can be put forward regarding either shared or side-specific factors.

First, working memory, which is strictly related to attentional control, is known to affect both MW (McVay & Kane 2009; Randall et al., 2014; Unsworth & McMillan, 2013) and RC (De Beni, et al., 1998; Follmer, 2018; Palladino et al., 2001). Low working memory capacity is associated with increased off-task thoughts (i.e., MW), which in turn might affect performance in attention-demanding tasks such as reading (McVay & Kane, 2012). However, low working memory is also recognized as a key component of reading comprehension process in itself, involving the ability to update relevant information and discard irrelevant ones (Palladino et al., 2001). This process results to be particularly relevant in building a situation model. When reading an easy text that abruptly becomes difficult by loading on working memory processes (e.g., longer sentences, low-frequency words), we should observe a decrement of MW and higher resources on comprehension performance. Conversely, someone with low working memory capacity would encounter either difficulty in comprehension processes (e.g., losing or being unable to detect relevant information) *and* lowered attentional control with increased MW*.* In other words, this person might be unable to dynamically adjust cognitive resources, resulting in high MW and low reading comprehension. A similar pattern might hold for the other moderators included in the present study, such as text difficulty and topic interest. Some previous studies suggested that MW susceptibility was not dependent on RC context (McVay & Kane, 2012) and that interest had an indirect effect on reading comprehension through MW (Unsworth & McMillan, 2013). In contrast, working memory capacity had both a direct effect on RC and an indirect one via MW, suggesting at least in some instances, MW has a causal influence on RC. For sure, the literature also reports a set of specific determinants that increase the likelihood of MW, and that might, indirectly, affect RC through MW. For instance, people who had reviewed their plans for the near future just before reading a text were more likely to engage in MW when reading (Kopp et al., 2015). However, most literature on the relationship between MW and RC comes from researchers who primarily investigated the effects of MW on RC, but less evidence has been collected on how RC can modulate MW. Therefore, we might develop a further proposal for future investigation—that is, to analyze if individual differences in the cognitive and linguistic processes that underlie RC might act as trigger for MW. In this view, we might hypothesize that individual differences in general cognitive functions used in reading (Li et al., 2022) play a role in the relationship between MW and RC.

RC is known to be affected both by decoding skills and linguistic abilities, according to the Simple View of Reading (Gough & Tunmer, 1986). People with reading disorders, for example, have been found to mind-wander more in self-paced reading compared with text-to-speech reading (Bonifacci et al., 2022). This suggests that alleviating the cognitive load associated with decoding in poor readers would allow them to be more on task, or, conversely, being involved in decoding increases the likelihood of engaging in MW. Therefore, poor attentional control and subsequent MW, in some instances, might be the result of decoding difficulties that overload on participants’ cognitive resources.

In other instances, a set of weaknesses in the comprehension process, as in the case of poor comprehenders (e.g., meta-analysis by Spencer & Wagner, 2018) might bring the mind to wander. Breakdowns in each step of the coFinally, difficulties in retrieving previous knowledge, lack of prior knowledgenstruction-integration model of reading comprehension would lead to an “overload” in terms of attentional control, which further enhances the mind’s chance to engage in MW.

For example, a poor vocabulary (Spencer et al., 2014) might interfere with the first surface level of text comprehension, letting the mind search for meanings and increasing the likelihood of off-task thoughts. Difficulties in grammar, morphology, and syntactic skills (e.g., Tong et al., 2014; Tong et al., 2011) might impact the construction of micro-and macro-structures at the proposition level and, in turn, detract resources from attentional control with an increased chance of MW in front of obstacles in RC. Finally, difficulties in retrieving previous knowledge, lack of prior knowledge, or difficulties in inferential processing might have a detrimental effect on the ability to build a situation model. When people fail to build a situational model, they are thought to disengage from the text, and mind wander (Kahmann et al., 2022).

Second, the strongest effect in moderation indices regards the methodological assessment of MW. Specifically, being trait-based questionnaires (i.e., when people are asked to report on their level of MW in daily life) used to assess MW instead of online probes (i.e., when people is required to respond to thought probes to assess their momentary MW while engaged in a reading task) would imply an average increase of 0.30 in the correlation between MW and text comprehension, thus leading to an almost null correlation. Conversely, both online and post self-report measures of MW produce similar results as probes (see Table 2). Although, in general, trait-based MW assessment and probes can be considered positively related (McVay & Kane 2009; Seli et al., 2016), the strength of this association is usually weak. In light of the results of our meta-analysis for which trait-based measures of MW tend to a null correlation with RC performance, one might question the validity of trait-based measures in reading research, as they may be only vaguely related to the complex network of processes linked to MW construct (e.g., Seli et al., 2016). With respect to our previously proposed theoretical approach, an absence of a relationship between trait MW and RC is in line with the idea that the swing effect might act only on the MW online process during the task: While state MW reflects momentary (transient) experiences, trait MW might be more related to a person’s personality and identity (da Silva, 2020). In this view, the two measures (i.e., state/trait) may capture only partially overlapping processes that may differently influence the effect of MW on RC. We did not find moderation effects regarding how RC was measured (i.e., multiple-choice vs. open-ended vs. true–false questions). However, the relatively low number of studies using open-ended questions makes it difficult to speculate on the possible reasons behind the lack of an effect. More research directly comparing performance in multiple-choice and open-ended questions would be useful to disentangle the relationship between MW and question type.

In summary, our proposal of a swing effect is complementary, rather than in contrast, with the cascade model of inattention (Smallwood, 2011), and suggests that there might be shared factors that influence both constructs simultaneously. In contrast, in other instances, there might be causal influences from one of the sides that indirectly affect the other side. Attentional control capacities might modulate how the reader is able to adjust the occurrence of MW while reading according to text characteristics and readers’ ability. Future studies should better understand shared and indirect (mediation) effects, taking account of both perspectives, including MW measures in RC studies and vice versa and possibly involving groups of participants with specific disorders in either attentional control (e.g., ADHD), decoding (e.g., dyslexia), or comprehension skills (e.g., poor comprehenders). It also has to be underlined that the relationship between RC and MW, although significant, is in the moderate range; therefore, the two constructs are only partially overlapping, and each of them might have independent features and pathways as well. Based on our results on MW assessment procedures, researchers should carefully interpret results from studies in which only trait-based measures have been employed to estimate the individual tendency to MW or to zoning out while reading.

There are some limitations in the present study that needs to be considered. First, in most analyses on moderators, the number of available data was limited and with limited, although acceptable, variability. In particular, more evidence is needed regarding the role of text interest and text difficulty. Further, other factors were not considered, such as the distinction between voluntary and involuntary MW, due to the absence of sufficient information in the selected studies. Finally, grey literature was not included in the meta-analysis, with potential overestimation of the reported effect. However, in this regard, given the small-to-medium correlation found and the absence of significant publication bias, the lack of gray literature does not appear to detract from the overall interpretation of the present study’s findings. Finally, we could not control precisely for section length, because this information was not reported consistently across studies; therefore, we considered the total number of words in the text, which was nonsignificant.

Despite these limitations, this is, to date, the first systematic analysis of the magnitude of the relationship between MW and RC. Current results are in keeping with the view that MW may be detrimental to RC, but the causal pathways that may determine such a relationship are yet to be documented.

Nevertheless, our results may have immediate practical implications. For instance, it is important to promote awareness of the relationship between MW and RC in educational settings. MW is not just a “distraction” from reading but rather a component of the process that might activate depending on both texts and participants’ characteristics. Developing strategies for reducing MW (e.g., mindfulness) might improve RC, and, on the other side, working on texts to favor RC (e.g., readability, topic interest) might reduce participants’ engagements in MW. A final issue that might open further research is related to the contents of MW. Given that MW, on certain occasions, has been found to have beneficial effects on cognition, further investigation should respond to the question about “which contents of MW interfere with RC and which are, instead, beneficial.”

## Data Availability

The synthesis of the literature reviewed in this study is openly available in Open Science Framework: https://osf.io/v835e/

## Code availability

The code used to perform the meta-analysis is openly available in Open Science Framework: https://osf.io/v835e/
